# A multi-bioassay integrated approach to assess antifouling potential of extracts from the Mediterranean sponge *Ircinia oros*

**DOI:** 10.1007/s11356-021-15683-8

**Published:** 2021-08-05

**Authors:** Lucia De Marchi, Carlo Pretti, Alessia Cuccaro, Matteo Oliva, Federica Tardelli, Gianfranca Monni, Michele Magri, Fabio Bulleri

**Affiliations:** 1 Dipartimento di Biologia – Unità di Ecologia e Biologia Marina, Università di Pisa, Pisa, Italy; 2 Consorzio per il Centro Interuniversitario di Biologia Marina ed Ecologia Applicata “G. Bacci” (CIBM), Livorno, Italy; 3 Dipartimento di Scienze Veterinarie, Università di Pisa, Via Livornese lato monte, 56122 San Piero a Grado (PI) Pisa, Italy; 4 Departamento de Biologia, Universidade de Aveiro, Aveiro, Portugal

**Keywords:** Sponge’ extract, Ecotoxicological screening, Bioassay, Antifouling, *Ficopomatus enigmaticus*, Development stages

## Abstract

The phylum Porifera and their symbionts produce a wide variety of bioactive compounds, playing a central role in their ecology and evolution. In this study, four different extracts (obtained by non-polar and semi-polar extraction methodologies) of the Mediterranean sponge *Ircinia oros* were tested through a multi-bioassay integrated approach to assess their antifouling potential. Tests were performed using three common species, associated with three different endpoints: the marine bacterium *Aliivibrio fischeri* (inhibition of bioluminescence), the marine diatom *Phaeodactylum tricornutum* (inhibition of growth), and different development stages of the brackish water serpulid *Ficopomatus enigmaticus* (gametes: sperm motion, vitality inhibition and cellular damage; larvae: development; adults: AChE (acetylcholinesterase)-inhibitory activity). The effects of extracts were species specific and did not vary among different extraction methodologies. In particular, no significant reduction of bioluminescence of *A. fischeri* was observed for all tested samples. By contrast, extracts inhibited *P. tricornutum* growth and had toxic effects on different *F. enigmaticus*’ developmental stages. Our results suggest that the proposed test battery can be considered a suitable tool as bioactivity screening of marine natural products.

**Supplementary Information:**

The online version contains supplementary material available at 10.1007/s11356-021-15683-8.

## Introduction

Marine sponges (Porifera) are an ancient metazoan group and a major component of benthic fauna throughout temperate, tropical, and polar environments (Bell 2008). Sponges are benthic sessile organisms, attached to rocky surfaces or shells, and are present both in open seas and estuaries (Châtel et al. 2011). These organisms provide key ecological services, including reworking of solid carbonate through bioerosion, nutrient recycling, primary production through microbial symbionts, clearance of the water column of prokaryotic plankton, and food for other biota (Wulff 2006). Sponges are active suspension feeders with the capability of absorbing ≈ 80% of suspended particles dissolved in the water column or present in sediments (Reiswig 1974; Müller and Müller 1998). By this mechanism, they are able to accumulate dissolved or suspended pollutants such as detergents (Zahn et al. 1977), polychlorinated biphenyls (PCBs) (Efremova et al. 2002), trace elements (Müller and Müller 1998; Rao et al. 2006; Cebrian et al. 2007; Gentric et al. 2016) polycyclic aromatic hydrocarbons (PAHs) (Batista et al. 2013), surfactants, organochlorines (Perez et al. 2002, 2003), organophosphorus, and carbamates (Marques et al. 2007).

In addition, sponges and their symbionts can exhibit chemical defence mechanisms against predation or epibiont overgrowth, often referred to as epibiosis or biofouling (Takur and Anil 2000; Tsoukatou et al. 2002; Hellio et al. 2005; Sánchez-Lozano et al. 2019). Biofouling on marine infrastructure, introduced to sustain a variety of maritime activities, including industry, shipping, energy production, fishing, or aquaculture, can cause substantial economic loss (Eguía and Trueba 2007; Hellio et al. 2005; Sánchez-Lozano et al. 2019; Vigneron et al. 2018). In order to reduce or slow down biofouling, paints based on different chemical forms of copper/zinc together with booster biocides (Eguía and Trueba 2007) have been and are still widely used. However, adverse environmental effects caused by these chemical compounds have been extensively demonstrated (Santillo et al. 2001; Thomas and Brooks 2010; Hasan et al. 2014; Batista-Andrade et al. 2018). Thus, the use of natural marine products able to reduce the recruitment and growth of fouling species on submerged structures may represent an innovative, nature-based solution to enhance the quality of marine urban ecosystems, such as marinas and ports.

Sponges represent a dominant component of Mediterranean benthic communities, with approximately 720 species (Costa et al. 2018). In particular, the genus *Ircinia* spp. is widely distributed on shallow rocky reefs in the Mediterranean Sea (Riesgo et al. 2016). This horny sponge, with a highly variable and irregular growth form, can reach up to 30 cm in diameter (Ledda et al. 2014). *Ircinia* spp. is a source of bioactive metabolites, such as linear or cyclic polyprenyl hydroquinones (Mihopoulos et al. 1999) and sesterterpenes (Cimino et al. 1972), many of which contain furan and tetronic acid functional groups. These metabolites can have antimicrobial and enzyme inhibitory activities (Duque et al. 2001; Thakur et al. 2004; Beedessee et al. 2013), as well as the ability to inhibit the growth of micro- and macro-algae (Tsoukatou et al. 2002) and the settlement of mussels, polychaetes, and barnacles (Tsoukatou et al. 2002; Hellio et al. 2005; Sánchez-Lozano et al., 2019).

In this study, two bioassays canonically used in ecotoxicological testing, namely inhibition of bioluminescence in *Aliivibrio fischeri* (bacteria) and inhibition of growth of *Phaeodactylum tricornutum* (diatom), were used to assess the toxicity of *Ircinia oros* extracts. Both organisms, considered as target model species, were used as surrogates to evaluate extracts potential to reduce the colonization of benthic biofilms, dominated by bacteria and diatoms (Koedooder et al. 2019; Caruso 2020). Antifouling potentials of *Ircinia oros* extracts were evaluated through an integrated approach, performing a series of assays at different development stages of the serpulid *Ficopomatus enigmaticus*, an invasive macro-fouler species able to colonize vessels and all submerged hard-substrata, forming massive reefs which removal implies considerable economic costs. In particular, we investigated how extracts affected different stages of this polychaete in terms of target (early stages) and non-target developmental stages (adult). Specifically, sperm motion and vitality, inhibition and cellular damage, larval development, and neuro-enzyme inhibitory activity at the adult stage were investigated. In addition, we assessed differences in the effectiveness of non-polar and semi-polar extraction.

## Material and methods

### Species collection

*Ircinia oros* specimens were collected at the end of September 2020 by SCUBA diving at 10-–12.5-m depth along the north-east coast of Elba Island (Nisportino, 42°50.160′N; 10°23.152′E, Italy; salinity 40‰, temperature 21 °C, pH 8.09). Two fragments (15×10 cm^2^ each) were inserted into a sterile plastic bag filled in with seawater and immediately transported to the laboratory. Samples were photographed in situ for better species characterization and a small piece was used for taxonomy confirmation.

### Preparation of extracts

Following Hellio et al. (2005), after collection, samples were rinsed with sterile seawater to remove associated debris. The surface microflora was removed by washing the sponge samples for 10 min with ethanol (30%) and then cut into small pieces, weighted, and freeze-dried. The methodology of extraction was performed based on Beedessee et al. (2013) and Tsoukatou et al. (2002), which is a specific method adopted for the genus *Ircinia* spp. Briefly, a first batch of dried sponges (20–40 g), obtained after 2-day oven-drying at 40°C, was macerated with MeOH/CH_2_Cl_2_ 1:1 for 24–48 h. After maceration, the solution was filtered and evaporated to dryness on a rotatory vacuum evaporator set at a temperature of 40 °C for few minutes. Distilled H_2_O was then added to the organic residue. The aqueous phases were collected, redissolved subsequently in hexane (non-polar extract) and then in ethylacetate (AcOEt) (semi-polar extract), and concentrated under vacuum at low temperature of 40 °C. A second batch of wet sponges (102–120 g) was macerated and partitioned in the same way as described for the first batch. The remaining organic residue containing non-polar and semi-polar extracts obtained from the same sample was resuspended in DMSO (10 mg/mL) and stored at −20 °C until the use. Stock solutions were then prepared in artificial seawater (ASW), according to ISO 10253 (2016). For each of the four extracts (dry sponges portioned with hexane (**DH**) and AcOEt (**DA**); wet sponges portioned with hexane (**WH**) and AcOEt (**WA**)), nominal concentrations were as follows: 100–50–25–10–5–1–0.5–0.25–0.1–0.05 μg/mL. For each extract, the range of concentrations was based on the following: (I) on a previous study by Hellio et al. (2005), and (II) to determine the limit of tolerance of each tested species, allowing the determination of EC_50_.

### Antifouling screening assays

The antifouling screening assays were performed through ecotoxicological evaluations using three common species associated with three different endpoints: the marine bacterium *Aliivibrio fischeri* (endpoint: inhibition of bioluminescence), the marine diatom *Phaeodactylum tricornutum* (endpoint: inhibition of growth), and the brackish water serpulid *Ficopomatus enigmaticus* at different development stages (gamete endpoints: sperm motion, vitality inhibition and cellular damage; larvae endpoint: larval development; adults endpoint: AChE (acetylcholinesterase)-inhibitory activity).

#### Inhibition of bioluminescence in *Aliivibrio fischeri*

The luminescent bacteria test was performed in accordance with the ISO 11348, 2007 methodology. The bacteria (strain n. 19A4002A, Ecotox LDS, Pregnana Milanese, MI, Italy) was purchased as freeze-dried bacterial cells. Dried bacteria were resuspended in 1 mL of reconstitution solution, acquired together with bacteria vials, in order to reactivate them. At first, a screening test was performed exposing reconstituted *A. fischeri’* suspensions to highest concentrations of all extracts (100 μg/mL) measuring differences in bioluminescence emission (percentage of bioluminescence inhibition – I %) with control (ASW) after 30 min of exposure. Ultraviolet filters showing an I % > 20% were investigated with a full test in order to calculate EC_10/20/50_. Then, *A. fischeri* bacteria were exposed to dilution series (see the “Preparation of extracts” section) (in ASW, salinity 30) and bioluminescence was again determined after 30 min of exposure. Tests were carried out in triplicate, at 15 °C, with sample pH within the operative range (6–8). All measurements were performed with a M500 luminometer, acquisition, and data processing (EC_10/20/50_ calculated by least square method) were performed with the Microtox® Omni 1.16 software. The reference toxicant was zinc sulfate eptahydrate (ISO 11348, 2007) and results obtained for this assay fell into the laboratory control chart (6.88–12.45 mg/L Zn^2+^).

#### Growth inhibition in *Phaeodactylum tricornutum*

The assessment of growth inhibition in *P. tricornutum* was performed following ISO procedures, with slight changes to the base protocol (ISO 10253, 2016). Briefly, *P. tricornutum* Bholin (CCAP 1052/1A) was the test strain and purchased from the reference center CCAP (Culture Collection of Alga and Protozoa Scottish Association for Marine Science/SAMS Research Services Ltd). Enriched saltwater medium (ASTM- ESM, ASTM E1218, 2012) was used for culturing *P. tricornutum* algae. The algal batch was prepared 72 h before the test, obtaining a logarithmic-phase algal culture. After 72 h, the batch was diluted to obtain a concentration of 10^6^ cell/mL. A sample of each sponge’ extract was diluted in ASTM-ESM to obtain all working concentrations, as reported in the “Preparation of extracts” section. Using 24-well plates (UNI-EN-ISO 10253, 2017), 20 μL of diluted algal batch were inoculated in each of three 2 mL replicates for both samples and controls (ASTM-ESM). Plates were left at 20 ± 2 °C, under continuous illumination (6000-8000 lx) and slow shaking (80 rpm) for 72 h. Growth after 72 h of exposure was the evaluated endpoint. Absorbance was measured spectrophotometrically at 670 nm (Abs670). Algal concentration (Cells×mL^−1^) was calculated from absorbance using the following equation:

$$ \mathrm{Cells}\ast {mL}^{-1}=\frac{Abs\ 670}{10^{-7}} $$

Values of EC_10/20/50_ for all extracts were calculated with the Linear Interpolation Method for Sublethal Toxicity software (U.S.EPA, 1993). The reference toxicant was potassium dichromate (ISO 10253, 2016), and results obtained for this assay fell into the laboratory control chart (1.94–4.36 mg/L Cr^2+^).

#### Assays on gametes, larvae, and adult in *Ficopomatus enigmaticus*

The brackish water serpulid. *F. enigmaticus* was collected in S. Rossore-Migliarino Regional Park – Fiume Morto (Pisa, Italy) at the end of October 2020. The reef was transferred to the laboratory covered in a wet towel. Water from the sampling site were used to set up the aquaria. In laboratory, the salinity was increased up to a maximum of 5 points/day, until reaching 30‰ (Oliva et al. 2018). During this period (temperature 22 ± 1 °C, oxygen saturation > 90%, salinity 30‰, pH 8.1 ± 0.1, photoperiod 10-h light:14-h darkness), organisms were daily fed with an *Isochrysis galbana* algal suspension (1×10^4^ cells/mL).

##### Gametes

The spawning of *F. enigmaticus* was induced mechanically by breaking the calcareous tube. According to Oliva et al. (2018), individuals were extracted from their tubes and placed one per well in a 24-well plate filled with 0.5 mL of ASW. The destructive method induced the gamete emission within 10 min. A pool of sperm suspensions were collected and put together in a sterile test tube and sperm concentration was determined by using a Burker counting chamber and an Olympus CH-2 optical microscope.

After gamete emission (Oliva et al. 2018), 200 μL of sperm suspension was incubated for 30 min at room temperature (RT) with 20 μL of tested concentrations (10-fold concentrated stock solutions) of each sponge’ extracts (see the “Preparation of extracts” section). The sperm motion assay was performed according to Elsayed et al. (2015), using the CASA plugin (computer-assisted sperm analysis system, University of California and Howard Hughes Medical Institute, USA) within the ImageJ software (a free open-source image processing software provided by the National Institutes of Health, http://imagej.nih.gov/ij/). Briefly, the incubated sperm suspensions were added with bovine serum albumin (BSA) 1% (v/v 1:1), placed on microscope glass slides and then examined under a Leica DMi1 inverted microscope, equipped with a MC120-HD camera, using a 40× magnification objective. The video camera recorded for 5 s (100 frame/s) the moving images (three videos per condition) of sperm cells and the computer digitized them. Digitized images consisted of pixels whose changing locations were recorded frame by frame after the exposure. The images were then processed using the CASA system. The values for input parameters in the CASA dialogue box were set according to a protocol developed by evo-devo-eco network (EDEN, http://edenrcn.com/), with modifications of the sperm size and FPS (number of frames/s) in order to accurately detect motile spermatozoa. CASA system performs its motion analysis through four main steps: (1) capturing image sequences of sperm, (2) object detection, (3) object tracking, and (4) calculation of motion characteristics such as curvilinear velocity (VCL), average path velocity (VAP), straight-line velocity (VSL), and linearity (LIN), which is the result of the rate between VSL and VAP; wobble (WOB), which is a measure of sperm head side to side movement and it is the result of the rate between VAP and VCL; progression (PROG) which represents the distance sperm travelled on VAP path; and beat cross frequency (BCF). The total percentage of sperms that were actively swimming (percentage of motility) were also calculated with CASA systems and the differences between control and exposed spermatozoids at each of the extraction method was performed.

A battery of sperm quality biomarkers (sperm vitality (MTT), lipid peroxidation (LPO), intracellular reactive oxygen species (ROS), and DNA damage) was adopted to assess the spermiotoxic effect of all tested extracts at all concentrations in *F. enigmaticus*, according to Cuccaro et al. (2020). Ten-fold concentrated stock solutions were prepared in ASW for each tested concentration (see the “Preparation of extracts” section). Analyses were performed adding 100 μL of extract to 1000 μL of sperm suspension for each concentration, which was incubated with for 30 min at room temperature (RT). After the exposure, the solutions were transferred on a 96-well plate for sperm quality assessment (Cuccaro et al. 2020).

##### Larvae

The larval development assay was performed according to Oliva et al. (2019). The numbers of normally or abnormally developed larvae were counted to calculate a percentage of poorly developed larvae. The acceptability threshold of the assay was set at 20% of poorly developed larvae in controls. The mean percentage of poorly developed larvae was calculated for each assessed concentration of all samples. EC_10/50_ values and their 95% confidence intervals were calculated via PROBIT analysis (Finney 1971).

##### Adults

Individuals of *F. enigmaticus* were extracted from portions of about 50 g of calcareous tubes (corresponding to ~ 80 individuals), weighted, and pooled in three replicates (80/90 individuals each). Each pooled sample was pulverized and then homogenized in a potter Elvejem in 100 mM phosphate buffer (pH 8, 1:2 w/v). Homogenates were centrifuged for 20 min at 9000 × *g* (S9) at 4°C and stored at −80 °C until used. AChE (acetylcholinesterase) activity was measured according to Ellman et al. (1961) in 100 mM phosphate buffer (pH 8.0), 10 mM DTNB, and 24 mM of substrates (ATChI). Enzyme activity was recorded continuously for 5 min at 412 nm at 25 °C in a BioTek Synergy HT micro-plate reader and the specific activity was corrected for the spontaneous hydrolysis of the substrate (nmol/min/mg protein). Protein content was determined according to the Lowry method (Lowry et al. 1951) using BSA as standard. For inhibition studies, aliquots of 200 μL of S9 fraction from each pool were incubated with each extract at different concentrations (100–50–25 and 10 μg/mL) for 30 min at 25° C before measuring the enzyme activities. The residual activity was determined as previously described. The concentration capable of inhibiting half of the enzyme activity (IC_50_ plus 95% confidence limits) was estimated for each extract.

### Statistical analyses

Results of ecotoxicological assays were reported as mean ± standard deviation (SD). Data were tested for normal distribution and homogeneity of variance by using the Kolmogorov-Smirnov and Bartlett’s tests, respectively. Since the two assumptions were met, the effects of sponge extract methodology and concentration were tested by means of a two-way ANOVA (GraphPad Prism version 6.00 for Windows, GraphPad Software, La Jolla California USA, www.graphpad.com).

## Results

### Inhibition of bioluminescence in *Aliivibrio fischeri*

Since there was no inhibition of *A. fischeri* bioluminescence in response to any of sponge’ extracts at tested concentrations (<12% of inhibition at 100 μg/mL for all tested toxicants), mean EC_10/20/50_ with respective 95% confidence limits are not reported.

### Growth inhibition in *Phaeodactylum tricornutum*

Sponges portioned with hexane (both dry and wet) induced lower EC_10/20/50_ values compared those portioned with AcOEt (Table 1). In details, DH presented the lowest EC_50_ (9.70 μg/mL) followed by WH (12.17 μg/mL). WA and DA extracts (EC_50_ of 38.26 and 42.99 μg/mL respectively) had weaker effects. Calculated values revealed the following increasing order of inhibition effects: DA > WA > WH > DH.

**Table 1:** Growth inhibition in *Phaeodactylum tricornutum*. Results are expressed as EC_10/20/50_ (μg/mL) together with 95% confidence limits (C.L.). ECs were obtained by a linear interpolation method. *n.c.* not calculable

Extracts	EC_10_	C.L. (95%)	EC_20_	C.L. (95%)	EC_50_	C.L. (95%)
**DA**	27.71	20.76–28.68	31.53	28.82–32.35	42.99	41.84–43.41
**WA**	8.22	6.95–10.0	16.85	10.33–20.75	38.26	36.86–40.38
**WH**	5.29	5.04–5.48	6.79	6.55–6.95	12.17	11.79–12.50
**DH**	0.79	0.46–1.07	6.09	4.08–5.27	9.70	9.02–10.56

*DA* dry sponges portioned with AcOEt, *WA* wet sponges portioned with AcOEt, *WH* wet sponges portioned with hexane, *DH* dry sponges portioned with hexane

### Assays on gametes, larvae, and adult in *Ficopomatus enigmaticus*

#### Gametes

As a consequence of high abnormal sperm morphology and mortality at 100, 50, and 25 μg/mL induced by all tested extracts, the estimation of sperm motion characteristics was performed from a range of concentrations of 10 to 0.5 μg/mL (Table 2). A dose-dependent decrease of all investigated sperm motions (VCL, VAP, VSL, LIN, WOB, PROG, and BCF) was caused by all tested extracts.

**Table 2:** Sperm motion assay in *Ficopomatus enigmaticus.* Results of sperm cells’ motion in microfluidic environments. *VCL* curvilinear velocity; *VAP* average path velocity; *VSL* straight-line velocity; *LIN* Linearity, VSL/VAP; *WOB* wobble (VAP/VCL), a measure of sperm head side to side movement; *PROG* progression (distance sperm traveled on VAP path); *BCF* beat cross frequency. The analysis was performed for all tested extracts (DH, DA, WH, WA) at the concentrations of 0.5, 1.0, 2.5, 5.0, and 10.0 μg/mL

Extracts	Concentrations (μg/mL)	VCL (μm/s)	VAP (μm/s)	VSL (μm/s)	LIN	WOB	PROG (μm)	BCF (Hz)
**DA**	0.0	350.16±167.80	167.35±124.63	392.37±61.18	1.82±1.40	0.44±0.14	3044.35±1914.31	25.31±6.69
	0.5	243.04±6.04	162.85±93.78	149.47±20.46	0.89±0.11	0.68±0.40	7175.25±3401.73	21.27±0.43
	1.0	161.69±12.97	76.63±0.89	64.76±0.17	0.85±0.01	0.48±0.03	2279.84±621.49	30.20±3.33
	2.5	192.46±31.07	143.97±52.51	133.81±49.73	0.93±0.01	0.74±0.15	4052.36±314.42	30.49±4.67
	5.0	169.21±45.43	106.50±60.98	90.28±64.05	0.81±0.14	0.60±0.20	2530.00±370.71	32.54±7.43
	10	199.54±53.54	63.49±20.43	67.41±39.03	1.02±0.29	0.32±0.02	1498.53±161.99	32.49±2.11
**WA**	0.0	281.25±63.57	122.73±61.81	316.94±63.48	2.10±1.90	0.42±0.12	2905.61±1295.90	32.60±2.91
	0.5	467.98±40.16	236.77±26.37	251.72±94.61	1.05±0.28	0.51±0.10	2122.46±70.16	21.35±0.48
	1.0	286.33±91.67	94.37±23.79	141.67±122.29	1.38±0.95	0.33±0.02	2026.11±483.93	28.84±2.76
	2.5	301.23±25.67	103.28±12.28	103.61±17.09	1.02±0.29	0.35±0.07	1651.09±344.56	29.42±1.52
	5.0	463.35±7.11	224.15±21.68	162.49±19.24	0.72±0.02	0.48±0.04	1945.81±716.41	25.61±1.89
	10	252.62±11.11	86.16±033	117.36±31.68	1.36±0.36	0.34±0.02	2133.28±416.21	30.64±2.08
**WH**	0.0	219.65±46.48	93.15±27.88	81.29±31.66	0.86±0.07	0.42±0.08	1920.20±822.18	28.80±6.10
	0.5	238.51±37.23	81.01±0.73	101.94±42.96	1.24±0.52	0.35±0.06	1408.17±209.45	30.25±0.64
	1.0	258.88±54.05	103.12±14.43	157.05±115.20	1.44±0.88	0.41±0.09	1963.98±789.30	29.65±1.79
	2.5	202.02±11.41	58.61±2.13	84.81±26.05	1.44±0.39	0.29±0.01	519.85±34.78	33.18±0.88
	5.0	252.76±37.78	137.72±60.67	149.32±48.50	1.11±0.14	0.53±0.16	4542.51±428.38	30.87±0.50
	10	231.09±22.14	85.77±5.81	98.38±49.70	1.11±0.50	0.37±0.06	1143.77±209.66	30.51±0.56
**DH**	0.0	257.97±11.04	105.07±68.22	241.88±40.66	1.97±1.01	0.39±0.10	1134.51±84.23	29.41±0.56
	0.5	296.99±96.65	89.47±22.22	164.48±77.92	1.79±0.43	0.31±0.02	1755.16±3.94	32.62±4.38
	1.0	299.53±29.37	93.75±9.68	249.08±27.49	2.80±2.02	0.31±0.06	1364.85±592.02	31.79±0.69
	2.5	569.60±190.40	375.11±293.89	286.85±126.22	0.76±0.02	0.61±0.31	3119.25±435.20	19.22±0.45
	5.0	383.63±26.96	106.07±4.43	83.82±14.56	0.79±0.10	0.28±0.01	1088.14±16.47	28.95±0.04
	10	255.88±115.38	59.02±15.20	111.40±68.00	2.11±1.69	0.24±1.69	1126.10±36.70	31.05±3.03

*DA* dry sponges portioned with AcOEt, *WA* wet sponges portioned with AcOEt, *WH* wet sponges portioned with hexane, *DH* dry sponges portioned with hexane

**Fig. 1:**
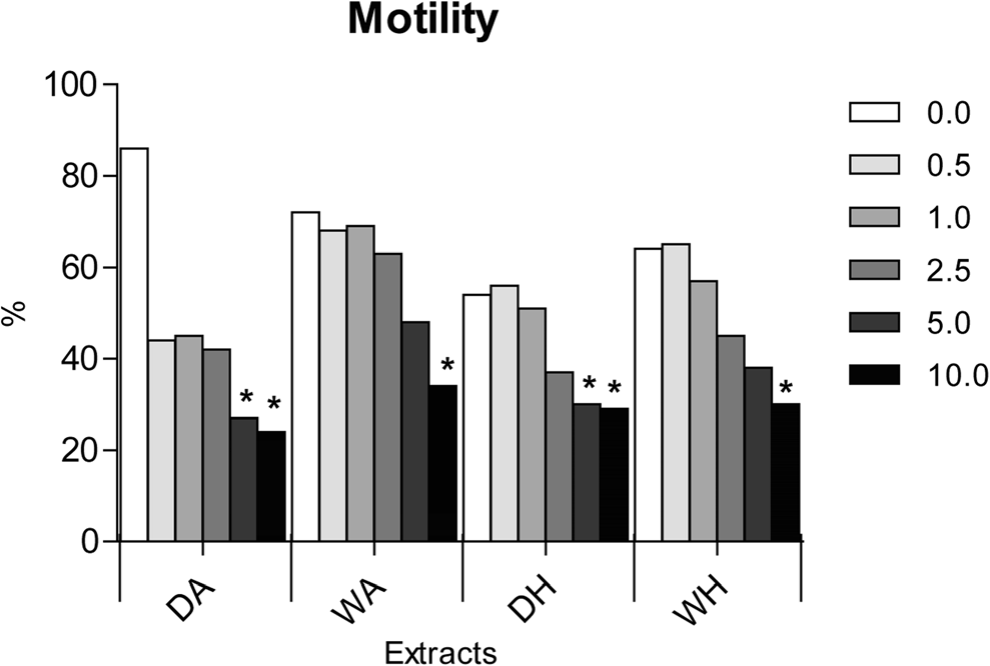
Results of sperm motility of *Ficopomatus enigmaticus* when exposed to a range of concentrations (0.0, 0.5, 1.0, 2.5, 5.0, and 10.0 μg/mL) of four different extracts (DA, dry sponges portioned with AcOEt; WA, wet sponges portioned with AcOEt; DH, dry sponges portioned with hexane; WH, wet sponges portioned with hexane). Results are expressed as a percentage. Mean ± SD; *p*< 0.05 (*); *p*< 0.01 (**)

The ANOVA showed that for the motility assay there was a significantly interaction between concentrations and extracts (15.24% of the total variance) as reported in Online Resource Table 1; nonetheless, only the concentration factor significantly affects the motility inhibition (Figure 1). In detail, DA and DH showed statistically lower values at both 10 and 5 μg/mL, while WA and WH only at 5 μg/mL compared to the respective controls (Figure 1).

**Fig. 2:**
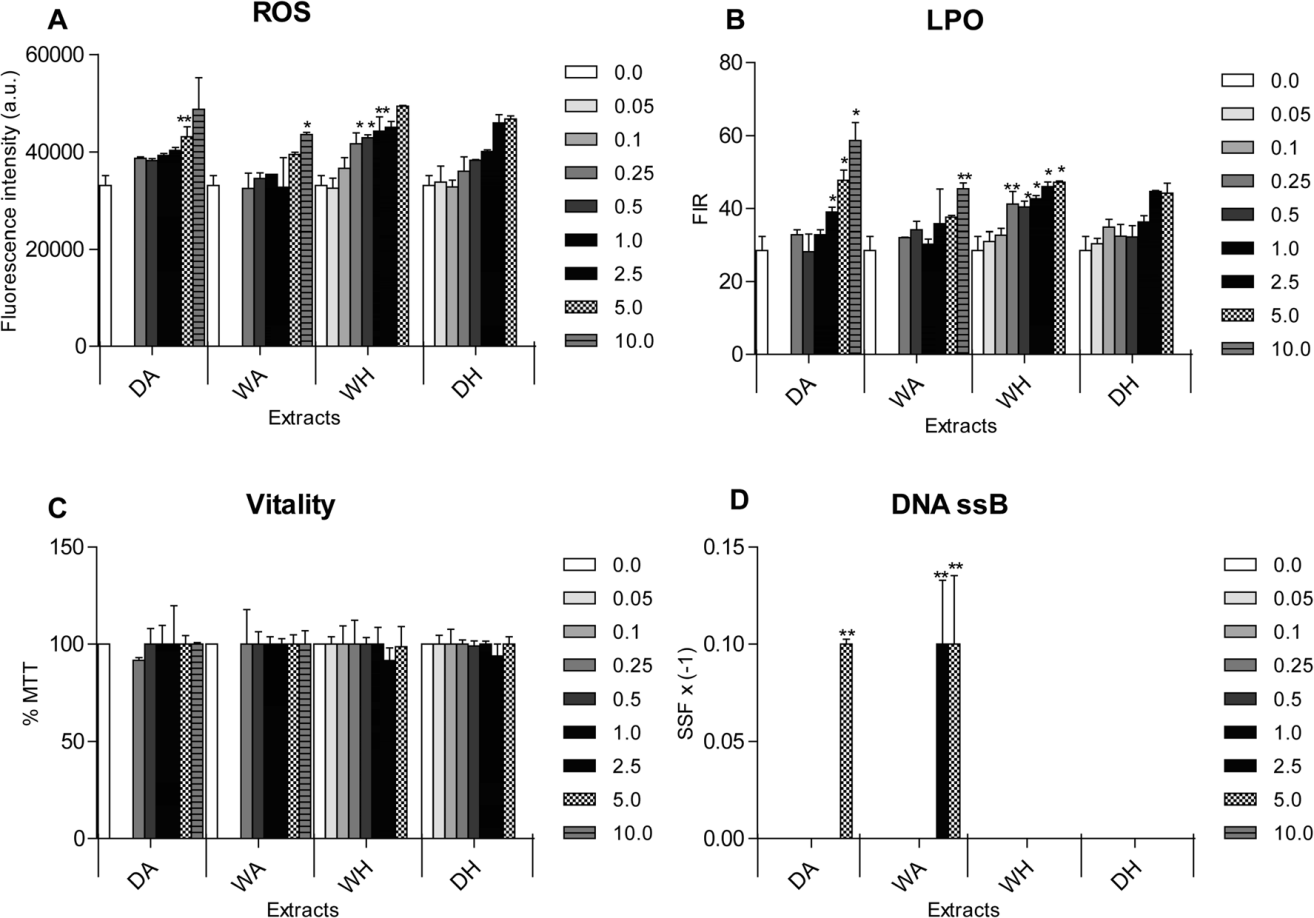
Results of spermiotoxicy tests on *Ficopomatus enigmaticus* when exposed to a range of concentrations (0.0, 0.05, 0.1, 0.25, 0.5, 1.0, 2.5, 5.0, and 10.0 μg/mL) of four different extracts (DA, dry sponges portioned with AcOEt; WA, wet sponges portioned with AcOEt; DH, dry sponges portioned with hexane; WH, wet sponges portioned with hexane). **A** Reactive oxygen species (ROS) levels were expressed as fluorescence intensity (a.u.); **B** Lipid peroxidation (LPO) mean levels were expressed as fluorescence intensity ratio (FIR); **C** Sperm vitality (MTT) mean values were expressed as percentage (%) of MTT; **D** Negative DNA Single-Strand-Break (DNA ssB) mean values were expressed as strand scission factors (SSF) x (−1) and the control corresponded to the x-axis. Mean ± SD; *p*< 0.05 (*); *p*< 0.01 (**)

Although there was an interaction between concentrations and extracts, sperm quality significantly varied only according to concentrations (data are given in Online Resource Table 2). Incubation of sperms with increasing concentrations of all extracts resulted in a progressive increase of ROS production, showing statistically higher values of sponges portioned with AcOEt (both dry and wet) at 5 and 10 μg/mL respectively and at concentrations ranging between 0.25 and 1.0 μg/mL for the WH (wet sponges portioned with hexane) treatment (Figure 2A).

A dose-dependent increase was also detected in terms of membrane peroxidation levels when sperms were exposed to all extracts, with statistically differences observed under DA, WA, and WH treatments. Specifically, for DA, significantly higher LPO levels were detected at 2.5, 5, and 10 μg/mL, for WA only at 10 μg/mL, and for WH at concentrations ranging between 0.25 and 5 μg/mL (Figure 2B).

The vitality of sperms (expressed as % of MTT) was not affected by any of tested extracts (Fig. 2C). Finally, there was a significant DNA damage at two highest concentrations of extracts, respectively, at 5 μg/mL for DA and both at 5 and 10 μg/mL for WA treatments (Figure 2D).

#### Larvae

Sponges portioned with hexane (both dry and wet) generally showed lower EC_10/50_ values compared the ones portioned with AcOEt (Online Resource Table 3). In detail, WH presented the lowest EC_50_ (0.89 μg/mL) followed by DH (1.69 μg/mL). WA and DA at EC_50_ of 1.21 and 5.12 μg/mL induced weaker effects on larval development, respectively. Calculated EC_50_ values revealed the following decreasing order of effects: DA > WA > DH > WH.

**Fig. 3:**
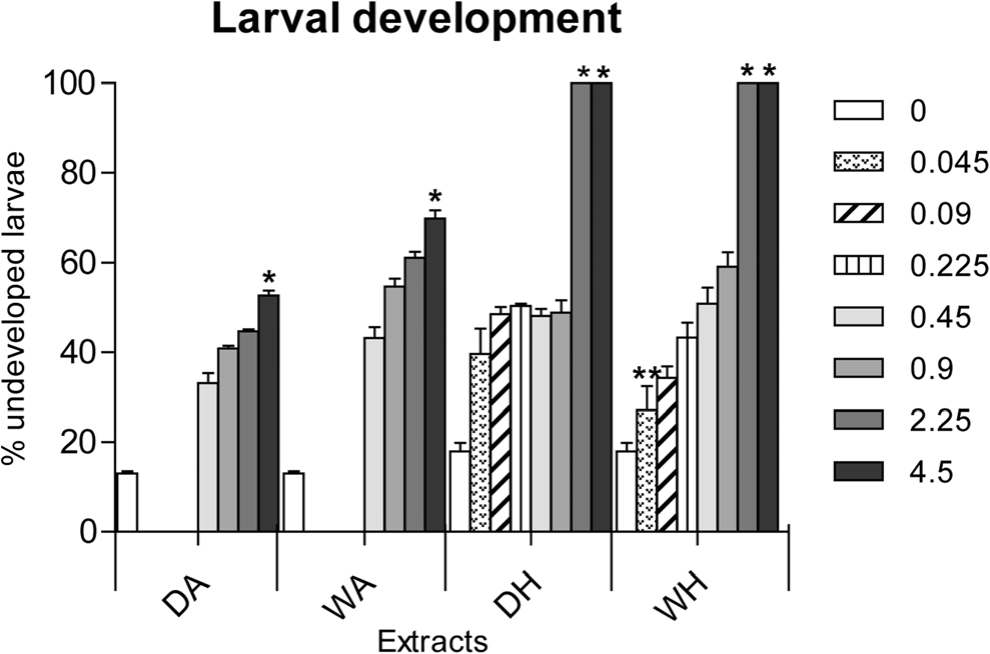
Results of *Ficopomatus enigmaticus* larval development assay when exposed to a nominal range of concentrations (0.0, 0.045, 0.09, 0.225, 0.45, 0.9, 2.25, and 4.5 μg/mL) of four different extracts (DA, dry sponges portioned with AcOEt; WA, wet sponges portioned with AcOEt.; DH, dry sponges portioned with hexane; WH, wet sponges portioned with hexane). Results are expressed as percentage of undeveloped larvae. Mean ± SD; *p*< 0.05 (*); *p*< 0.01 (**)

The ANOVA showed significant effects of extract concentrations on the proportion of larva successfully developed, consistently among extracts. Data are reported in Online Resource Table 4. Differences were significant between control and the highest concentration (DA and WA) and between the two highest concentrations (DH and WH), with successful larval development decreasing as concentrations increased. Moreover, all sponge extracts, when at 10 μg/mL, caused a 100% death of larvae (Figure 3).

#### Adults

**Fig. 4:**
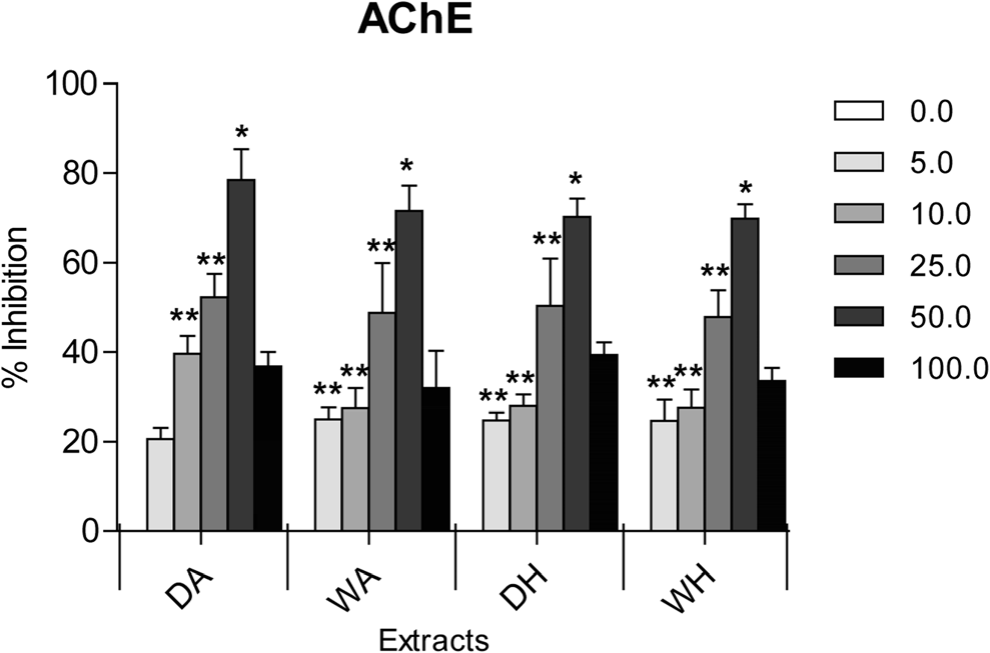
Results of *Ficopomatus enigmaticus* AChE (acetylcholinesterase)-inhibitory activity when exposed to a range of concentrations (0.0, 5.0, 10.0, 25.0, 50.0, and 100.0 μg/mL) of four different extracts (DA, dry sponges portioned with AcOEt; WA, wet sponges portioned with AcOEt; DH, dry sponges portioned with hexane; WH, wet sponges portioned with hexane). Results are expressed as a percentage. Mean ± SD; *p*< 0.05 (*); *p*< 0.01 (**)

Since there was no neurotransmitter inhibition of *F. enigmaticus* fractions incubated at concentrations ranging between 0.05 and 1.0 μg/mL of all extracts, the estimation of the inhibitory activity on the enzyme was examined from 5 to 100 μg/mL. Inhibition of the AChE activity varied significantly with extract concentration (Online Resource Table 5). Increasing extract concentrations significantly raised the percentage of inhibitory effects compared with their respective controls (Figure 4).

## Discussion

Four different extracts obtained from the sponge *I. oros* were tested investigating their potential antifouling purposes. The extraction methods adopted were selected following Beedessee et al. (2013), who observed that AcOEt extracts of the sponges *Pericharax heteroraphis* and *Amphimedon navalis* displayed competitive/non-competitive inhibition associated with terpenoids and steroids (Beedessee et al. 2013). Several studies have shown that sponges are rich in terpenoids and steroids, which can be effective in reducing predation rates (Pawlik 2012) competition for space (Duque et al. 2001; Pawlik et al. 2007; Helber et al. 2018) and epibiosis (Thakur and Singh 2016). Specifically, Hellio et al. (2005) found that extracts (the mixture of ircinin I and II sesterterpenes) from epibiont-free specimen of the Mediterranean sponge *I. oros* were highly active in inhibiting the settlement of the barnacle *Balanus amphitrite* (i.e., higher percentage of swimming larvae and lower percentage of settling larvae).

Sponges have been extensively used as growth bacterial inhibitors. Mol et al. (2009) demonstrated that acetone extracts from sponges of the genus *Haliclona* spp. inhibited the growth of several bacteria, including *Bacillus cereus*, *B. pumilus*, *B. megaterium*, *Pseudoalteromonas haloplanktis*, *Pseudomonas chlororaphis*, *P. putida*, and *P. aeruginosa.* In this study, none of tested extracts reduced the bioluminescence in the marine bacteria *A. fischeri* in comparison with controls. Although this bioassay has been widely applied for evaluating compound toxicity since it is cost-effective and easy to use (Abbas et al. 2018), a number of studies have reported a low sensitivity for toxicity measurement of some chemicals such as antibiotics and mycotoxins (Abbas et al. 2018).

Different results were obtained testing the same extracts on other target species. All four extracts had a calculable EC_10/20/50_ on the growth of *P. tricornutum*. Negative effects were stronger for non-polar (with hexane) than semi-polar extracts (AcOEt). Significant bioactivity on microalgae inhibition has also been reported for other screening strategies, such as extracting *I. oros* metabolites with a polar solvent (ethanol) (Tsoukatou et al. 2002), suggesting their effectiveness in inhibiting microalgal growth.

One of the main concerns about the use of natural chemical compounds is the impact that they can have on invertebrate organisms (Hasson et al. 2011). Considering that the effects of any toxicant can vary across life stages (Mohammed 2013), the effectiveness of sponge’ extracts was evaluated at different stages of *F. enigmaticus* both in terms of target (early stages) and non-target developmental stages (adult). Although marine invertebrate gametes and larvae are several orders of magnitude more sensitive than adults, assessing the sensitivity at the adult stage is necessary for determining the effects of a compound throughout a species life cycle (Holan et al. 2018).

In general, all *I. oros*’ extracts showed negative effects on sperm kinematics (motion characteristics), even if not concentration dependent. Many substances extracted from marine organisms have been shown to have a profound influence on the reproductive behaviour and strategy’s function (Silvestre and Tosti 2009). Considering the percentage of sperm progressive motility, negative concentration-dependent effects were caused by all extracts. Sperm motility is highly dependent on several metabolic pathways and regulatory mechanisms. Any abnormality in these factors could be responsible of poor sperm motility and one of the main causes was already identified in biochemical deficits of sperms (Pereira et al., 2017). To validate this hypothesis, we assessed a battery of biomarkers related to sperm damage (LPO, ROS production and DNA damage) and vitality (MTT). Once again, there was a significant dose-dependent increase in ROS production compared to controls, especially at highest exposure concentrations. Reactive oxygen species (ROS), such as the superoxide anion (O_2_^−^), hydrogen peroxide (H_2_O_2_), and nitric oxide (NO^−^), are chemically reactive molecules resulting from oxygen consumption. At certain concentrations, ROS are of extreme importance to sperm function (Agarwal et al. 2003; Ford 2004). Specifically, some studies have demonstrated the involvement of ROS in sperm functionality, reporting that NO^−^ induce capacitation at low levels while it blocks sperm motility at high levels. In addition, also high O_2_^−^ and H_2_O_2_ levels can be deleterious for sperm functionality affecting motility parameters (Herrero et al. 1999; O’Flaherty et al. 1999; Du Plessis et al. 2010; Pereira et al., 2017). In this study, the excessive ROS production was also confirmed by (I) the dose-dependent oxidative damage of cellular membranes caused by all sponge extracts and (II) the DNA damage observed at highest concentrations of DA and WA extracts (dry and wet *I. oros* portioned with AcOEt). As a matter of fact, the imbalance in the rate of ROS generation leads to oxidative stress. The products of lipid peroxidation chain reactions display high biological activity destroying DNA, proteins, and enzymes as well as activating signaling pathways which cause cell death (Su et al. 2019). Our results could be partly driven by the high abnormal sperm morphology and mortality that emerged at 100, 50, and 25 μg/mL and with the consequent use of low concentrations of sponge’ extracts, this hypothesis can justify the obtained. However, although the integrity of the membrane and the damage of DNA were significantly correlated with sperm kinematics and motility, as they were related to an increase of excessive ROS, no variation in terms of sperm vitality was detected for all extracts under all exposed concentrations compared to their relative controls.

In this work, the larval development assay as a target developmental stage in ecotoxicological studies was also used as endpoint of extract toxicity. The selected assay was focused on the first developmental stage, which brings zygotes to a planktonic and planctotrophic trocophore stage (Oliva et al. 2018, 2019). Because the first larval development endpoints are commonly considered of high sensitivity to a large number of chemical substances (Mohammed et al.,^ 2009^), embryotoxicity tests have become more common. All four extracts showed a statistically significant effect in terms of inhibition of larval development with calculable EC_50/10_ values. However, although no statistical differences were obtained among the different extracts, the sensitivity of larvae to hexane (both dry and wet) was greater (lower EC_50/10_ values) than that to AcOEt extracts. The toxicity assessments of natural mixtures and emerging contaminants were recently applied to *F. enigmaticus* (Oliva et al. 2018, 2019, 2020), confirming the sensitivity of this organism as well as the embryotoxicity test as a valuable tool in developmental toxicology. Our results are in line with those of Herath et al. (2019), who, using extracts from two marine sponges (*Monanchora unguiculata* and *Haliclona* sp.), found significant effects on the development of larvae of the nematode, *H. contortus*; in, particular, a dose-dependent inhibition of the motility of third-stage larvae as well as of the development of the fourth-stage larvae.

The relationship between reduced settlement and development inhibition in sessile organisms generated by sponge extracts, through the alteration of neurotransmitter enzymes activity, has been previously demonstrated (Garaventa et al. 2010). In details, Garaventa et al. (2010) observed that expression of poly-alkylpyridinium salts (poly-APs), a mixture of two polymers obtained from the Mediterranean sponge *Haliclona sarai*, was able to generate specific and non-toxic acetylcholinesterase (AChE) inhibition in vitro. The substance was first tested for its effect on larval development of *A. Amphitrite* confirming the ability of Poly-APs to prevent the development of sessile organism by impinging on AChE activity. Our results are in agreement with previous evidence of sponge extract-induced neurotransmitter inhibition, confirming that AChE is an enzyme associated to the cholinergic signal system, and is also involved in cell-to-cell communication, driving embryonic development (Wessler and Kirkpatrick 2008). Sensitivity to natural antifouling compounds in the adult stages (non-target) of marine invertebrates was also demonstrated by Simons et al. (2021), who found *Acartia tonsa* adult females to be more sensitive to bacteria-produced algicide compared to its nauplii stage.

## Conclusion

The topic of the present work was based on an ecotoxicological evaluation through a multi-bioassay integrated approach to assess the effectiveness of *Ircinia oros*’ extracts as potential antifouling in terms of biology responses. All extracts showed species-specific effects without differences among extraction methodologies. In details, no significant reduction of bioluminescence in *A. fischeri* assay was observed for all tested samples. In contrast, all tested extracts had a significant bioactivity on microalgal inhibition, as well as toxic effects on target and non-target developmental stages of *F. enigmaticus*.

Thus, in order to pursuing the use of sponge’s extracts as natural-based antifouling booster compounds confirming their effectiveness, further evaluations appear warranted. Although different studies already demonstrated antifouling effects caused by specific metabolites extracted from the genus *Ircinia* spp., further assays are mandatory to individuate those responsible for the observed toxicity and, ultimately, attempt their active use in new-generation antifouling paints.

## Supplementary information

ESM 1 (DOCX 19 kb)

## Data Availability

All data generated or analyzed during this study are included in this published article [and its supplementary information files].
