# Supplementary material for: A multi-bioassay integrated approach to assess antifouling potential of extracts from the Mediterranean sponge Ircinia oros
Source: Environ Sci Pollut Res Int. 2021 Aug 5;29(1):1521–31. doi: 10.1007/s11356-021-15683-8 (PMC8724186; doi:10.1007/s11356-021-15683-8)
Supplement: Supplementary file 1 — (DOCX 19 kb) [file 11356_2021_15683_MOESM1_ESM.docx]

**Table 1.** *Ficopomatus enigmaticus*: Motility assay: Two-way ANOVA output for motility assay performed for all tested extracts (DH, DA, WH, WA) at the respective concentrations (0.5; 1.0; 2.5; 5.0 and 10.0 µg/mL). The analysis was followed by a post-test (Bonferroni) for multiple comparison. Source of variations, Df, F and significances (*p* < 0.001) were reported.

| **Motility** | | | | |
| --- | --- | --- | --- | --- |
| Source of variation | % tot variation | Df | F | Significant? |
| Interaction | 15.24 | 15 | 108200 | yes |
| Concentrations | 69.68 | 5 | 1475000 | yes |
| Extracts | 14.98 | 3 | 528600 | no |
| Residual |  | 48 |  |  |

**Table 2.** *Ficopomatus enigmaticus*: Vitality and Cellular damage: Two-way ANOVA output for sperm quality biomarkers (intracellular reactive oxygen species (ROS)**,** lipid peroxidation (LPO), sperm viability (MTT) and DNA damage) performed for all tested extracts (DH, DA, WH, WA) at the respective concentrations (0.05; 0.1; 0.25; 0.5; 1.0; 2.5; 5.0 and 10.0 µg/mL). The analysis was followed by a post-test (Bonferroni) for multiple comparison. Source of variations, Df, F and significances (*p* < 0.001) were reported.

| **ROS** | | | | |
| --- | --- | --- | --- | --- |
| Source of variation | % tot variation | Df | F | Significant? |
| Interaction | 50.67 | 24 | 40.88 | yes |
| Concentrations | 41.61 | 8 | 100.7 | yes |
| Extracts | 3.99 | 3 | 25.78 | no |
| Residual |  | 72 |  |  |
| **LPO** | | | | |
| Source of variation | % tot variation | Df | F | Significant? |
| Interaction | 55.35 | 24 | 26.10 | yes |
| Concentrations | 35.44 | 8 | 50.14 | yes |
| Extracts | 2.85 | 3 | 10.74 | no |
| Residual |  | 72 |  |  |
| **MTT** | | | | |
| Source of variation | % tot variation | Df | F | Significant? |
| Interaction | 55.23 | 24 | 26.15 | yes |
| Concentrations | 36.29 | 8 | 51.83 | yes |
| Extracts | 1.94 | 3 | 7.35 | no |
| Residual |  | 72 |  |  |
| **DNA ssB** | | | | |
| Source of variation | % tot variation | Df | F | Significant? |
| Interaction | 44.90 | 24 | 9.27 | yes |
| Concentrations | 31.08 | 8 | 19.26 | yes |
| Extracts | 9.50 | 3 | 15.69 | no |
| Residual |  | 72 |  |  |

**Table 3.** *Ficopomatus enigmaticus:* Larval development assay: Results are expressed as EC_10/50_ (µg/mL) together with 95 % confidence limits (C.L.). ECs were obtained by the use of a PROBIT model. n.c: not calculable because badly developed larvae were always under 20%.

| Extracts | EC_10_ | C.L. (95%) | EC_50_ | C.L. (95%) |
| --- | --- | --- | --- | --- |
| **DA** | 3.66 | 2.58-4.40 | 5.12 | 4.23-5.85 |
| **WA** | 0.14 | - | 1.21 | - |
| **WH** | 0.027 | 0.001-0.079 | 0.89 | 0.46-3.28 |
| **DH** | 0.002 | 0.00-0.021 | 1.69 | 0.52-104.84 |

**DA**: dry sponges portioned with AcOEt; **WA**: wet sponges portioned with AcOEt.; **WH**: wet sponges portioned with hexane; **DH**: dry sponges portioned with hexane

**Table 4.** *Ficopomatus enigmaticus*: Larval development assay: Two-way ANOVA output for larval development assay performed for all tested extracts (DH, DA, WH, WA) at the respective concentrations (0.05; 0.1; 0.25; 0.5; 1.0; 2.5; 5.0 and 10.0 µg/mL). The analysis was followed by a post-test (Bonferroni) for multiple comparison. Source of variations, Df, F and significances (*p* < 0.001) were reported.

| **Larval development** | | | | |
| --- | --- | --- | --- | --- |
| Source of variation | % tot variation | Df | F | Significant? |
| Interaction | 6.82 | 33 | 46.11 | yes |
| Concentrations | 86.22 | 11 | 1750 | yes |
| Extracts | 6.54 | 3 | 486.4 | no |
| Residual |  | 96 |  |  |

**Table 5.** *Ficopomatus enigmaticus*: AChE-inhibitory assay: Two-way ANOVA output for AChE-inhibitory assay performed for all tested extracts (DH, DA, WH, WA) at the respective concentrations (5.0; 10.0; 25.0; 50.0 and 100.0 µg/mL). The analysis was followed by a post-test (Bonferroni) for multiple comparison. Source of variations, Df, F and significances (*p* < 0.001) were reported.

| **AChE-inhibitory** | | | | |
| --- | --- | --- | --- | --- |
| Source of variation | % tot variation | Df | F | Significant? |
| Interaction | 1.13 | 15 | 0.40 | no |
| Concentrations | 89.39 | 5 | 95.24 | yes |
| Extracts | 0.47 | 3 | 0.83 | no |
| Residual |  | 48 |  |  |
